# Multi-class segmentation of knee MRI based on hybrid attention

**DOI:** 10.3389/fmed.2025.1581487

**Published:** 2025-06-11

**Authors:** Yuhang Xiang, Xinglin Zhang, Tao Meng, Tao Chen

**Affiliations:** ^1^School of Medical Information Engineering, Gannan Medical University, Ganzhou, China; ^2^Shanghai Medical Image Insights Intelligent Technology Co., Ltd., Shanghai, China; ^3^Jiangxi Rimag Group Co., Ltd., Nanchang, China; ^4^Big Data Research Lab, University of Waterloo, Waterloo, ON, Canada; ^5^Labor and Worklife Program, Harvard University, Cambridge, MA, United States

**Keywords:** medical image segmentation, deep learning, attention mechanism, knee, MRI

## Abstract

**Introduction:**

Accurate segmentation of knee MRI images is crucial for the diagnosis and treatment of degenerative knee disease and sports injuries. However, many existing methods are hindered by class imbalance and fail to capture the features of small structures, leading to suboptimal segmentation performance.

**Methods:**

This study applies hybrid attention and multi-scale feature extraction methods to the problem of multi-class segmentation of knee MRI images and innovates the classic U-Net architecture. Firstly, we propose a Hierarchical Feature Enhancement Fusion (HFEF) module, which is integrated into both the skip connections and the bottleneck layer. This module captures channel and spatial information at multiple levels, enabling the model to efficiently combine local and global features. Secondly, we introduce the Atrous Squeeze Attention (ASA) module, which enables the model to focus on multi-scale features and capture long-range dependencies, thereby improving the segmentation accuracy of complex multi-class structures. Lastly, the loss function is optimized to address the challenges of class imbalance and limited data. The improved loss function enhances the model's ability to learn underrepresented classes, thus enhancing the overall segmentation performance.

**Results:**

We evaluated the proposed method on a knee MRI dataset and compared it with U-Net. HASA-ResUNet achieved a 12.12% improvement in Intersection over Union (IoU) for the low-frequency and small-sized class, the anterior cruciate ligament, and a 3.32% improvement in mean Intersection over Union (mIoU) across all classes.

**Conclusion:**

These results demonstrate that the proposed hybrid attention and multi-scale strategy can effectively address the challenges of class imbalance in knee MRI images, improving the model's overall segmentation performance.

## 1 Introduction

Osteoarthritis (OA) is the dominant form of degenerative musculoskeletal disease, impacting approximately 5% of the worldwide population (1). The knee is the most frequently invaded site of OA (2). The prevalence of knee osteoarthritis (KOA) is especially high among the elderly, leading to severe pain, functional impairment, and limited mobility, which significantly reduce patients' quality of life (3–5). With further research, KOA has been recognized as a chronic joint disease involving structures such as articular cartilage, subchondral bone, and surrounding soft tissues, all of which directly affect knee joint mobility (6). Therefore, achieving early diagnosis and accurate assessment of KOA is of crucial importance.

There are numerous types of osteoarticular diseases, and more than 70% of diagnoses require medical imaging examinations. X-Ray and Computed Tomography have received broad attention from doctors because of low cost and high efficiency. However, they rely heavily on density differences to form images, making them less effective at discriminating soft tissues. In comparison, MRI provides comprehensive imaging of various structures and is widely used in the diagnosis and evaluation of KOA. Furthermore, it is considered the most effective non-invasive method for quantitative morphological assessment of knee cartilage due to its high accuracy (7).

Segmenting knee joint structures is essential for measuring the desired functional parameters in MRI images, and it has received considerable attention. In practice, it is time-consuming and labor-intensive to segment the anatomical structures of the knee manually, so automatic segmentation of knee images has a strong practical demand in the clinic. Notably, Convolutional Neural Networks (CNN) have demonstrated remarkable capabilities in feature extraction and information representation, and have become a hot research topic in the field of medical image segmentation. In 2013, Prasoon et al. (8) proposed using deep learning to segment tibial cartilage and utilized a triplanar convolutional neural network by combining three 2D CNNs. Although only 2D features were used, the three-plane CNN still outperformed a state-of-the-art method based on 3D features in segmentation accuracy. Liu et al. (9) developed and evaluated a new musculoskeletal segmentation algorithm that combined SegNet with 3D simplex deformable modeling to refine the segmentation results, preserving the anatomical structures' shape while smoothing tissue boundaries. The UNet-CGAN model (10) employed adversarial training and incorporated Dice and cross entropy losses into the loss function, effectively guiding the training process of the generator. The model achieved a Dice Similarity Coefficient (DSC) of 0.87 and 0.89 for the medial and lateral meniscus, respectively, and an average DSC of 0.88 for cartilages. Chen et al. (11) proposed a network structure similar to pix2pix, which consists of a generator for generating masks and a discriminator for distinguishing the produced masks from the true labels. Furthermore, by introducing adversarial loss, this method significantly improved the segmentation performance of knee bone and cartilage, with a validation score exceeding 76 on the SKI10 dataset (12). Woo et al. (13) developed a multi-step method for the initial detection of abnormalities in the distal femur, proximal tibia, and patella in individuals with varying degrees of KOA. Subsequently, the extracted data were used for downstream segmentation tasks. The anomaly-aware network demonstrated higher sensitivity and specificity. However, research on full knee joint structures segmentation is still very limited due to the scarcity of medical annotation data. Based on 3D fast spin-echo (FSE) sequence images, Zhou et al. (14) effectively achieved accurate segmentation of 12 types of knee joint structures by integrating CNN, 3D fully connected conditional random field (CRF), and 3D simplex deformable modeling. Although this study exhibited good performance on 3D-FSE images with good tissue contrast, it remains necessary to explore the clinical potential and applicability of 2D-FSE images.

**Figure 1:**
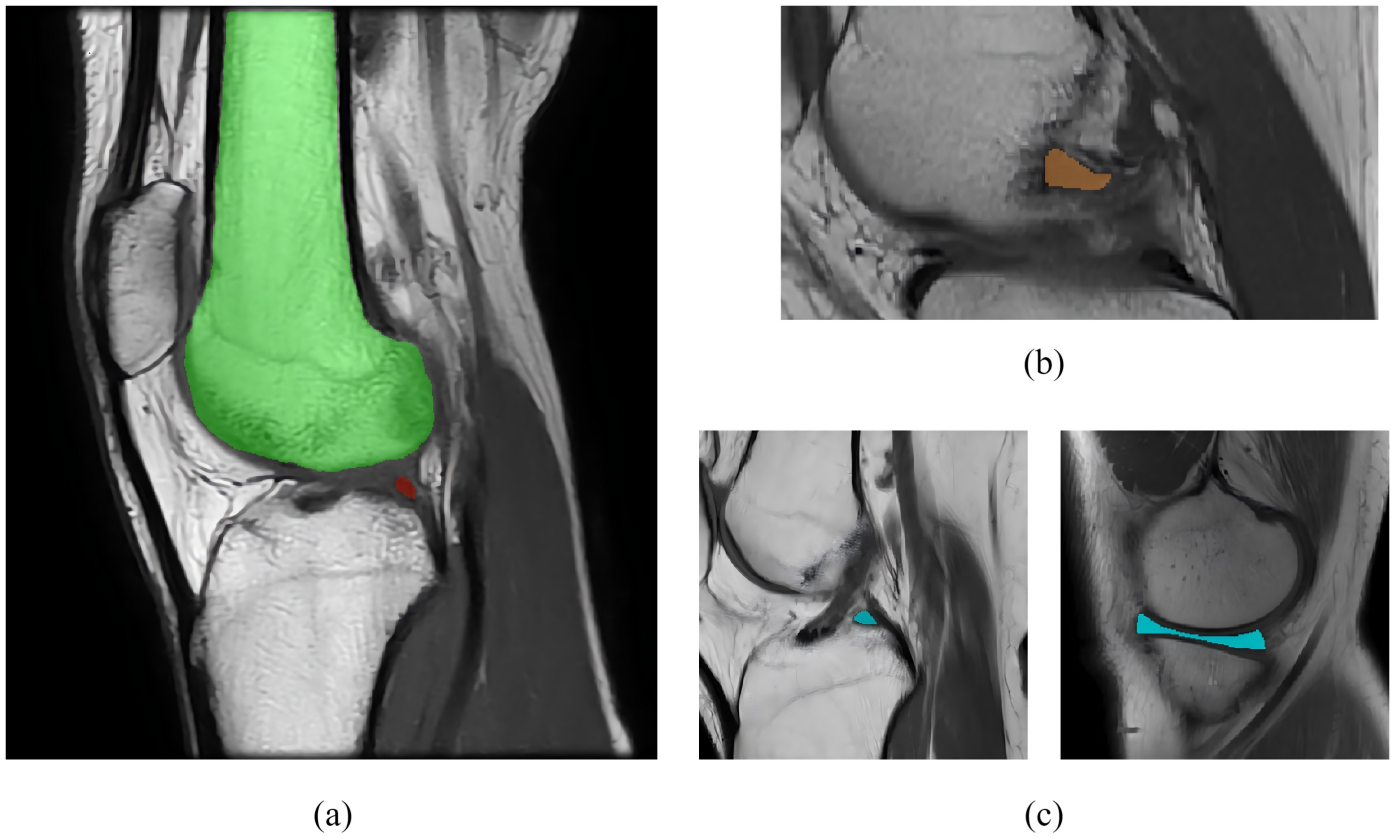
**(a)** Pixel imbalance. **(b)** Blurred boundaries. **(c)** Shape diversity.

To date, automatic segmentation of knee MRI images remains challenging, primarily due to three reasons, as shown in Figure 1. Challenge 1: Pixel imbalance. In knee MRI images, certain small structures (e.g., the meniscus) occupy significantly fewer pixels compared to larger structures like bones, as illustrated in Figure 1a. When neural networks are trained on class imbalance datasets, they are prone to overfitting the training samples of underrepresented classes, which may result in poor generalization during testing (15). Challenge 2: Blurred boundaries. The complexity and low contrast of the anatomy within the joint cavity make it challenging to achieve accurate localization and segmentation, which can easily lead to false positives or false negatives, as depicted in Figure 1b. Challenge 3: Shape diversity. The knee contains multiple types of tissues (e.g., meniscus, ligaments, bones, etc.), which exhibit significant variations in shape, size, and position across different layers, as shown in Figure 1c. Therefore, achieving optimal performance on both large and small anatomical structures is a challenging task.

In this paper, we propose HASA-ResUNet, a novel network designed to address challenges in multi-class anatomical segmentation of the knee joint, which comprises two key modules: a Hierarchical Feature Enhancement Fusion (HFEF) module with hybrid attention, and an Atrous Squeeze Attention (ASA) module. HFEF is introduced between the low-level and high-level stages of the model. It extracts rich contextual features by leveraging channel and spatial attention mechanisms, embedding them into high-level representations to enhance feature fusion. The ASA is located in the final layer of the decoder, where it comprehensively captures and integrates multi-scale features in the image, thereby improving the quality of output details. Consequently, in terms of the average DSC across all structures, our model outperforms other U-Net variants. Specifically, our contributions are as follows:

- To address the challenge of effectively capturing small structures with the network, we employ the HFEF module, which enhances the fusion of high-resolution spatial information from shallow layers and rich semantic information from deep layers. This approach effectively preserves fine structural details and improves the model's ability to distinguish the boundaries of small anatomical structures.
- To tackle the difficulty of segmenting knee joint tissues with diverse shapes, sizes, and complex boundary relationships, we introduce the ASA module. By introducing atrous pyramid convolution, this module enables the model to focus on multi-scale feature representations and capture long-range dependencies, thereby strengthening its ability to perceive features at different scales and significantly improving the segmentation accuracy of multi-class tissues.
- Considering the class imbalance in multi-class segmentation tasks, we introduce a hybrid loss function to enhance the network's learning ability for under-represented classes and improve the robustness of the training process.

In our experiments, we evaluated the performance of HASA-ResUNet in segmenting knee joint structures in MRI images. The results demonstrated that HASA-ResUNet improved the accuracy of knee segmentation, especially on small structures.

## 2 Materials and methods

### 2.1 Dataset

The image dataset consists of 163 sagittal T1-weighted FSE knee cases, with approximately 15–25 images per sequence, resulting in a total of 2,910 slices. These images were acquired at 1.5T and 3.0T using scanners from all major MR vendors. The data was randomly divided into training, validation, and test sets in a ratio of 8:1:1. A multi-class mask was developed for every image, containing the following value mappings: 0 = background, 1 = skin, 2 = femur, 3 = tibia, 4 = lateral meniscus, 5 = medial meniscus, 6 = patella, 7 = patellar ligament, 8 = fibula, 9 = posterior cruciate ligament, and 10 = anterior cruciate ligament. The annotations were performed manually by three radiologists, with special attention paid to the accuracy of the target structures and surrounding tissue boundaries during the annotation process. In the training process, we applied online data augmentation techniques, including random rotation, Gaussian noise, elastic deformation, and brightness augmentation. These techniques introduced random perturbations to the original data to generate new training samples, thereby increasing the diversity of the dataset. However, intensity non-uniformity can arise from variations in acquisition sources, imaging devices, and scanning parameters, which may significantly impact the consistency and reliability of image analysis across scans. Therefore, we standardized the raw image intensities prior to analysis. Specifically, we applied z-score normalization, adjusting each image to have a mean intensity of 0 and a standard deviation of 1. This transformation ensured consistency in image processing and minimized intensity variations between patient scans, improving the robustness of subsequent analysis.

### 2.2 The network architecture of HASA-ResUNet

**Figure 2:**
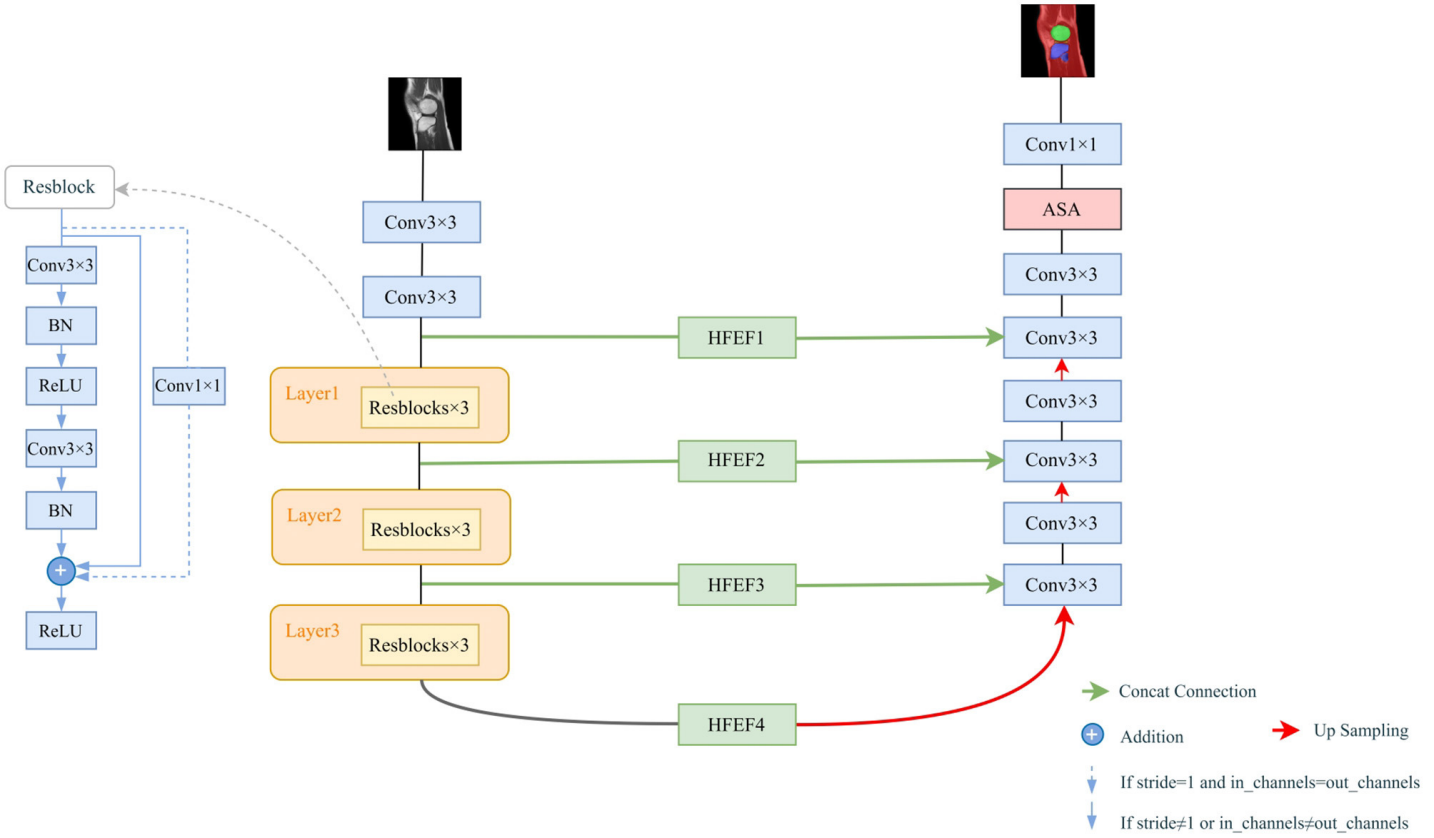
The structure of the HASA-ResUNet. Residual connections are added in encoding blocks.

The overall architecture of our proposed segmentation model is shown in Figure 2. It is a U-shaped encoder-decoder network. The model consists of three core components: the encoder, the decoder, and the skip connections. Based on ResNet (16), we proposed using residual blocks to replace the original convolutional layers in U-Net. Residual units enable the construction of deeper neural networks by effectively mitigating the vanishing gradient problem. In contrast, the decoder still uses the traditional convolutional layers. After each upsampling operation, feature maps of the same scale from the corresponding feature extraction part are concatenated along the channel dimension. However, the shallow network features primarily describe structural details, which differ from the high-level semantic features in the upsampling path. Direct concatenation may negatively impact subsequent processing. To address this, we designed a novel bridging structure using the HFEF module, which significantly improves segmentation accuracy and feature fusion by suppressing redundant information and alleviating semantic mismatches. Additionally, before generating the final segmentation results, we introduced the ASA module to enhance the model's ability to capture multi-scale features and long-distance dependencies, thus improving the segmentation accuracy of multi-class structures.

### 2.3 Hierarchical feature enhancement fusion

**Figure 3:**
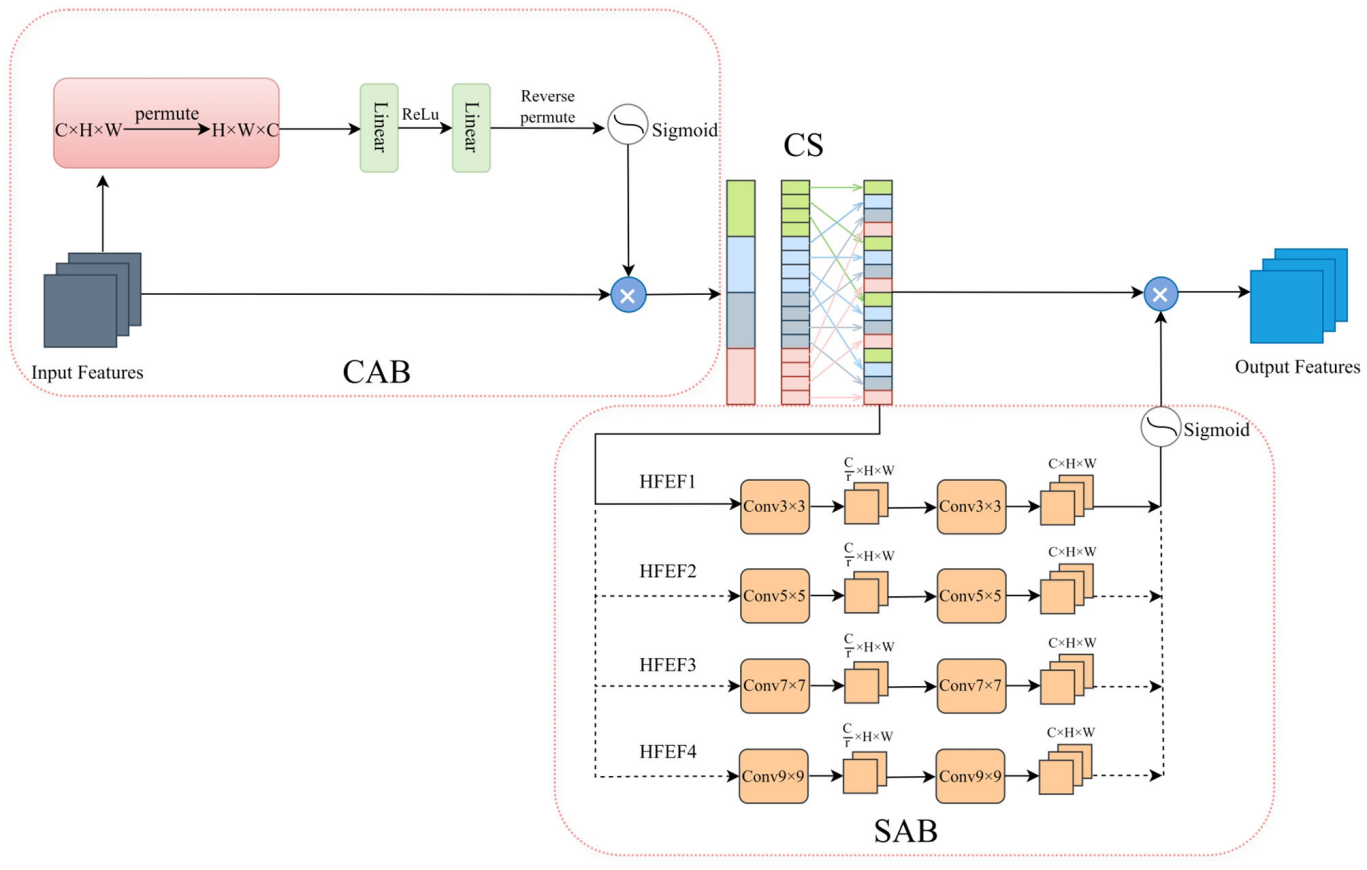
The structure of the HFEF. The components from left to right are CAB, CS, and SAB. HFEF1, HFEF2, HFEF3, and HFEF4 represent different hierarchical levels of the HFEF structure.

It is challenging to accurately identify the boundaries of small structures, such as the meniscus and ligaments. These structures often exhibit low contrast and irregular shapes, making it difficult for models to effectively capture spatial details. To address this problem, we introduced the Hierarchical Feature Enhancement Fusion (HFEF) module, strategically placed between the encoder and decoder stages of the network. As shown in Figure 3, the core architecture of the HFEF module consists of three components: the Channel Attention Block (CAB), the Channel Shuffle (CS), and the Spatial Attention Block (SAB). The CAB and SAB serve as complementary attention mechanisms to capture inter-channel dependencies and spatial relationships, respectively. This design adaptively recalibrates the feature maps and highlights important task-relevant information to enhance segmentation performance for small and complex anatomical structures.

The CAB recalibrates channel-wise features by modeling inter-channel dependencies. Let the input feature map be denoted as *X* ∈ ℝ^*C*×*H*×*W*^, where *C*, *H*, and *W* represent the number of channels, height, and width, respectively. The process can be formalized as follows:

1. **Permutation:** Reorganize the input feature map from *X* ∈ ℝ^*C*×*H*×*W*^ to $X_{p}\in {\mathbb{R}}^{H\times W\times C}$ to facilitate channel-wise operations.
2. **Channel compression and expansion:**
  - Apply a linear layer to reduce the channel dimension:
    $$Z_{1}=W_{1}X_{p}+b_{1},\;Z_{1}\in {\mathbb{R}}^{H\times W\times \frac{C}{4}}$$
    where $W_{1}\in {\mathbb{R}}^{\frac{C}{4}\times C}$ and $b_{1}\in {\mathbb{R}}^{\frac{C}{4}}$ are the weights and bias, respectively.
  - Introduce nonlinearity with ReLU:
    $$Z_{2}=\text{ReLU}\left(Z_{1}\right)$$
  - Restore the original channel dimension with a second linear layer:
    $$Z_{3}=W_{2}Z_{2}+b_{2},\;Z_{3}\in {\mathbb{R}}^{H\times W\times C}$$
    where $W_{2}\in {\mathbb{R}}^{C\times \frac{C}{4}}$ and $b_{2}\in {\mathbb{R}}^{C}$.
3. **Permutation back:** Reshape *Z*_3_ back to $Z_{4}\in {\mathbb{R}}^{C\times H\times W}$.
4. **Channel attention map:** Apply a sigmoid activation to generate attention weights:
  $$M_{c}=\sigma \left(Z_{4}\right),\;M_{c}\in {\mathbb{R}}^{C\times H\times W}$$
  where σ denotes the sigmoid function.
5. **Feature recalibration:** Element-wise multiplication with the input:
  $$X_{c}=M_{c}⊙X$$
  where ⊙ denotes element-wise multiplication.

To further mix and share information, a channel shuffle operation is subsequently applied. The enhanced feature maps are divided into four groups, each containing C/4 channels. Then, a transpose operation is performed on the grouped feature maps to shuffle the channel order within each group. Afterward, the shuffled feature maps are restored to their original shape (C × H × W). This approach enhances the interaction between channels, balances the feature representation, and effectively integrates global and local features. Given $X_{c}\in {\mathbb{R}}^{C\times H\times W}$, The process can be formalized as follows:

1. **Group division:** Split *X*_*c*_ into *S* = 4 groups along the channel dimension:
  $$X_{c}=\left[G_{1},G_{2},G_{3},G_{4}\right],\;G_{i}\in {\mathbb{R}}^{\frac{C}{4}\times H\times W}$$
2. **Transpose and shuffle:** Rearrange the channels within and across groups. This can be modeled as a permutation function π:
  $$X_{s}=\pi \left(X_{c}\right),\;X_{s}\in {\mathbb{R}}^{C\times H\times W}$$
  where π interleaves channels from different groups (e.g., taking one channel from each group cyclically).

Successful segmentation relies on the effective combination of local and global contextual information. Low-level features contain rich spatial details, while high-level features provide advanced semantic information (17). Given the importance of low-level features for small targets, small convolutional kernels are used in the lower layers to better extract fine-grained details. Simultaneously, larger convolutional kernels are used for high-level semantic information to capture global contextual information. Specifically, the four hierarchical levels of the SAB employ convolutional kernels of sizes 3×3, 5×5, 7×7, and 9×9, respectively, to capture spatial dependencies at different scales. This enables the extraction of rich and effective feature combinations, thereby helping the model to accurately locate and segment target structures. Given $X_{s}\in {\mathbb{R}}^{C\times H\times W}$, the SAB process can be represented by the following equations:

1. **Multi-scale convolution:** Apply convolutional kernels of sizes 3 × 3, 5 × 5, 7 × 7, 9 × 9 at different hierarchical levels (e.g., HFEF1 uses 3 × 3). For a kernel size *k* × *k* (e.g., *k* = 3):
  - Reduce channels:
    $$F_{1}={\text{Conv}}_{k\times k}\left(X_{s},W_{k,1}\right),\;F_{1}\in {\mathbb{R}}^{\frac{C}{4}\times H\times W}$$
    where $W_{k,1}\in {\mathbb{R}}^{\frac{C}{4}\times C\times k\times k}$.
  - Apply Batch Normalization (BN) and ReLU:
    $$F_{2}=\text{ReLU}\left(\text{BN}\left(F_{1}\right)\right)$$
  - Restore channels:
    $$F_{3}={\text{Conv}}_{k\times k}\left(F_{2},W_{k,2}\right),\;F_{3}\in {\mathbb{R}}^{C\times H\times W}$$
    where $W_{k,2}\in {\mathbb{R}}^{C\times \frac{C}{4}\times k\times k}$.
  - Apply BN:
    $$F_{4}=\text{BN}\left(F_{3}\right)$$
2. **Spatial attention map:** generate weights with a sigmoid:
  $$M_{s}=\sigma \left(F_{4}\right),\;M_{s}\in {\mathbb{R}}^{C\times H\times W}$$
3. **Feature recalibration:** apply the spatial attention:
  $$X_{\text{HFEF}}=M_{s}⊙X_{s}$$

The final output *X*_HFEF_ combines channel and spatial attention, enhancing feature fusion across scales.

### 2.4 Atrous squeeze attention

**Figure 4:**
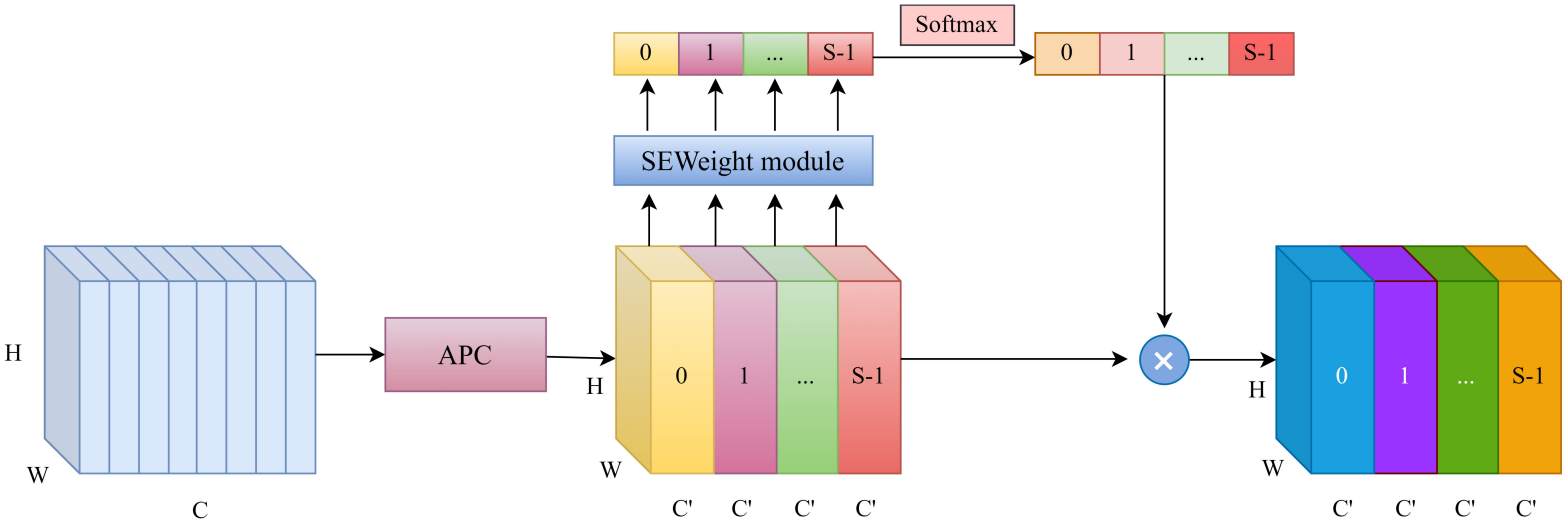
The structure of the ASA.

In traditional U-Net, successive convolution and max-pooling operations often lead to the neglect of small structures in multi-class segmentation tasks. To effectively capture spatial information at various scales, a multi-scale feature extraction method is adopted to enhance the model's perceptual capability. Specifically, the Atrous Squeeze Attention (ASA) module integrates pyramid atrous convolutions and a channel attention mechanism, as shown in Figure 4.

**Figure 5:**
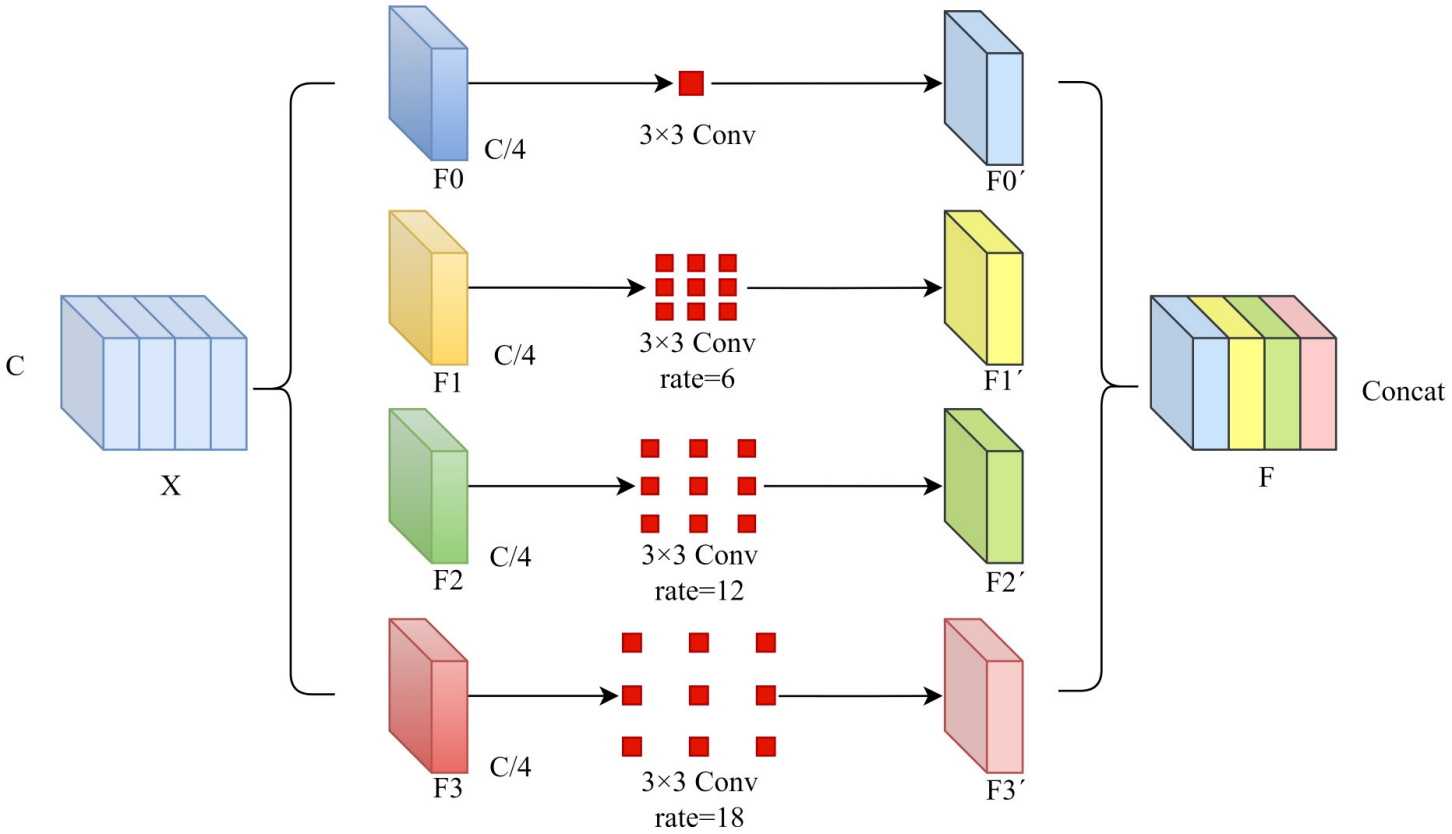
The structure of the proposed Atrous Pyramid Concat (APC) module.

The original Squeeze Pyramid Concat (SPC) (18) module can generate feature representations with different spatial resolutions and depths through the use of multi-scale pyramid convolution kernels. However, as the convolution kernel size increases, the number of parameters also grows significantly. Inspired by Atrous Spatial Pyramid Pooling (ASPP) (19), we improved the original SPC module into Atrous Pyramid Concat (APC) as shown in Figure 5. We adopted a parallel atrous convolution method to extract multi-scale information, which not only maintains a larger receptive field but also reduces computational load, making it more efficient. The input channel dimension is C. By compressing the channel dimension of the input tensor, each set of feature maps Fi is assigned a uniform channel dimension $C^{\prime }=\frac{C}{S}$ (S represents the number of groups, and here S=4), enabling efficient extraction of spatial information across different scales. All preprocessed feature maps are spliced in a concatenation way, where F denotes the resulting multi-scale feature maps. Then, channel attention weights are computed to emphasize informative features across different scales. The ASA module is mainly implemented in four steps. First, APC is used to extract spatial information at different scales from each channel-wise feature map. Second, the Squeeze-and-Excitation (SE) module is utilized to capture inter-channel correlations by adaptively adjusting the channel weights. Third, softmax is applied to recalibrate the attention vector. Finally, the recalibrated weights are applied to the corresponding feature maps through element-wise multiplication, resulting in attention-enhanced multi-scale feature representations. The ASA module enhances multi-scale feature extraction using atrous convolutions and channel attention. It is placed in the decoder's final layer. Let the input feature map be *X* ∈ ℝ^*C*×*H*×*W*^. The APC module consists of the following components.

1. **Channel compression:** Split *X* into *S* = 4 groups:
  $$X=\left[F_{0},F_{1},F_{2},F_{3}\right],\;F_{i}\in {\mathbb{R}}^{\frac{C}{4}\times H\times W}$$
2. **Atrous convolution:** Apply atrous convolutions with different dilation rates *r* (e.g., *r* = 1, 6, 12, 18) to each group:
  $$F_{i}^{\prime }={\text{AtrousConv}}_{3\times 3}\left(F_{i},W_{i},r_{i}\right),\;F_{i}^{\prime }\in {\mathbb{R}}^{\frac{C}{4}\times H\times W}$$
  where $W_{i}\in {\mathbb{R}}^{\frac{C}{4}\times \frac{C}{4}\times 3\times 3}$ and *r*_*i*_ is the dilation rate for group *i*.
3. **Concatenation:** Combine the multi-scale features:
  $$F=\text{Concat}\left(F_{0}^{\prime },F_{1}^{\prime },F_{2}^{\prime },F_{3}^{\prime }\right),\;F\in {\mathbb{R}}^{C\times H\times W}$$

The SE mechanism recalibrates channel weights as follows:

1. **Global average pooling:** Compress spatial dimensions:
  $$z=\frac{1}{H\times W}\overset{H}{\underset{h=1}{\sum }}\overset{W}{\underset{w=1}{\sum }}F\left(:,h,w\right),\;z\in {\mathbb{R}}^{C}$$
2. **Channel excitation:**
  - Reduce dimensionality:
    $$z_{1}=W_{1}z+b_{1},\;z_{1}\in {\mathbb{R}}^{\frac{C}{4}}$$
  - ReLU:
    $$z_{2}=\text{ReLU}\left(z_{1}\right)$$
  - Restore dimensionality:
    $$z_{3}=W_{2}z_{2}+b_{2},\;z_{3}\in {\mathbb{R}}^{C}$$
3. **Attention weights:** Apply softmax to normalize weights:
  $$M_{a}=\text{Softmax}\left(z_{3}\right),\;M_{a}\in {\mathbb{R}}^{C}$$
4. **Feature recalibration:** Scale the feature map:
  $$X_{\text{ASA}}=M_{a}\cdot F$$
  where *M*_*a*_ is broadcasted across spatial dimensions.

### 2.5 Loss function

The loss function plays a critical role in the training process by guiding the model's learning, optimizing the parameters, and ultimately influencing its performance. Most loss functions in image segmentation tasks are based on cross-entropy or coincidence measures. Traditional CE loss treats all classes equally. Specifically, the CE loss evaluates the divergence between the predicted probability distribution and the ground truth, demonstrating strong performance when class distributions are balanced. However, class imbalance remains a persistent challenge in semantic segmentation, particularly in medical imaging applications. Relying solely on CE loss during training can result in a model that is disproportionately biased toward the majority class. Dice loss was introduced in V-Net (20), which penalizes the spatial overlap difference between predicted and true annotations. This encourages the model to focus more on small classes, making it particularly effective for imbalanced datasets. We used a hybrid loss function that combines CE loss and Dice loss to address the challenge of unbalanced training data in multi-class segmentation of the knee. CE loss considers each pixel as an independent sample, while Dice loss evaluates the final prediction output in a more holistic way. The loss function is expressed as follows:


(1) $$\begin{array}{l} \text{CE Loss}=-\frac{1}{N}\overset{N}{\underset{i=1}{\sum }}\left[y_{i}\log\left({\hat{y}}_{i}\right)+\left(1-y_{i}\right)\log\left(1-{\hat{y}}_{i}\right)\right] \end{array}$$

where *N* is the total number of samples, *y*_*i*_ is the true label of sample *i*, and ${\hat{y}}_{i}$ is the predicted probability that sample *i* belongs to foreground.


(2) $$\begin{array}{l} \text{Dice Loss}=1-\frac{2\sum_{i=1}^{N}y_{i}{\hat{y}}_{i}}{\sum_{i=1}^{N}y_{i}+\sum_{i=1}^{N}{\hat{y}}_{i}} \end{array}$$

The Dice loss is defined as one minus the Dice score. The Dice score, a widely used metric for pixel-wise segmentation, is adapted in this manner to serve as a loss function.


(3) $$\begin{array}{l} \text{Loss}=w_{1}\times \text{CE Loss}+w_{2}\times \text{Dice Loss} \end{array}$$

where *w*_1_ and *w*_2_ are adjustable parameters used to balance the values of the two loss functions. In the experiment, *w*_1_ and *w*_2_ are set to 0.5.

## 3 Experiment and results

### 3.1 Implementation details

**Figure 6:**
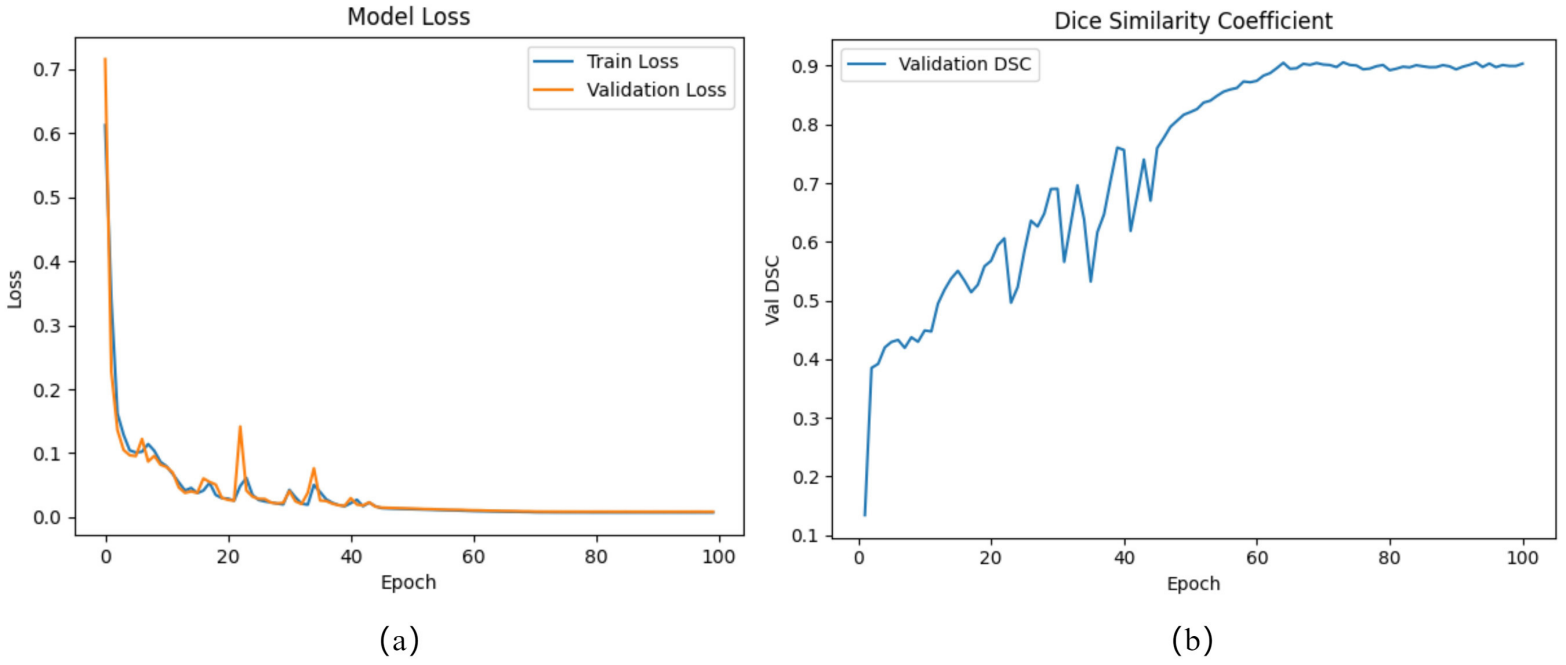
**(a)** Train and validation loss curves. **(b)** Validation DSC curve.

All experiments were conducted using the PyTorch 2.0.0 framework and were run on a single NVIDIA GeForce RTX 4090 GPU. The model was trained with an image resolution of 512 × 512. In the training process, the learning rate was set to 1 × 10^−4^, and the model was trained over 100 epochs with a batch size of 6 and optimized using Adam. To prevent overfitting and improve training efficiency, early stopping was employed during model training. Training was stopped if the DSC on the validation set did not improve for 5 consecutive epochs. Figures 6a, b depict the loss curves and the validation DSC curve, respectively.

### 3.2 Evaluation metrics

Taking the segmentation of the femur as an example, a pixel is called true positive (TP) if it is correctly assigned to the femur and is defined as false negative (FN) if it is misclassified to some other category. A background pixel is called true negative (TN) if it is correctly categorized and is defined as false positive (FP) if it is misclassified as a femur pixel. Four image segmentation evaluation metrics were utilized in this study, including Dice Similarity Coefficient (DSC), Intersection over Union (IoU), precision, and recall.

DSC is the most commonly used metric in image segmentation tasks, especially for measuring the overlap between two sets, such as predicted and ground truth segmentations. The DSC is defined as:


(4) $$\begin{array}{l} \text{DSC}=\frac{2\times TP}{2\times TP+FP+FN} \end{array}$$

To calculate the IoU score for each class, divide the intersection between the ground truth and the predicted segmentation by the union of the ground truth mask and the predicted segmentation mask. The IoU is calculated as follows:


(5) $$\begin{array}{l} \text{IoU}=\frac{TP}{TP+FP+FN} \end{array}$$

Precision measures how many of the samples predicted as positive belong to the positive class. It is the ratio of the number of correctly predicted positive samples to the total number of samples predicted as positive.


(6) $$\begin{array}{l} \text{Precision}=\frac{TP}{TP+FP} \end{array}$$

Recall measures how many of the actual positive samples are correctly predicted as positive. It is the ratio of the number of correctly predicted positive samples to the total number of actual positive samples.


(7) $$\begin{array}{l} \text{Recall}=\frac{TP}{TP+FN} \end{array}$$

### 3.3 Comparative experiments

We used DeepLabv3+ (21), ResUNet++ (22), U-Net (23), HRNet (24), UNet-VGG16 (25), and UNet-ResNet50 (26) to validate the performance on the knee dataset and compared the results with our model. Our model achieved the best performance, significantly outperforming the U-Net and its representative variants. Compared to U-Net, our model improved DSC, IoU, precision, and recall by 2.46%, 3.32%, 1.46%, and 2.58%, respectively. Table 1 shows the final results of different models on the test set. Our model not only outperforms other models across various metrics, but the visualization results highlight its exceptional overall performance in the multi-class segmentation task.

**Table 1:** Metrics of our model compared with DeepLabv3+, ResUNet++, U-Net, HRNet, UNet-VGG16, and UNet-ResNet50 models on the knee joint dataset.

**Methods**	**DSC**	**IoU**	**Precision**	**Recall**	**Trainable params(M)**	**Inference time(s)**
DeepLabv3+	0.8423	0.7632	0.8666	0.8394	39.63	0.65
ResUNet++	0.8534	0.7760	0.8683	0.8538	4.06	0.27
U-Net	0.8745	0.7985	0.8987	0.8667	31.04	0.63
HRNet	0.8589	0.7842	0.8769	0.8508	28.54	0.25
UNet-VGG16	0.8780	0.8080	0.8994	0.8677	24.89	0.66
UNet-ResNet50	0.8549	0.7801	0.8824	0.8449	43.93	0.29
**Ours**	**0.8991**	**0.8317**	**0.9133**	**0.8925**	32.98	0.89

Bold values indicates the highest metric values.

**Figure 7:**
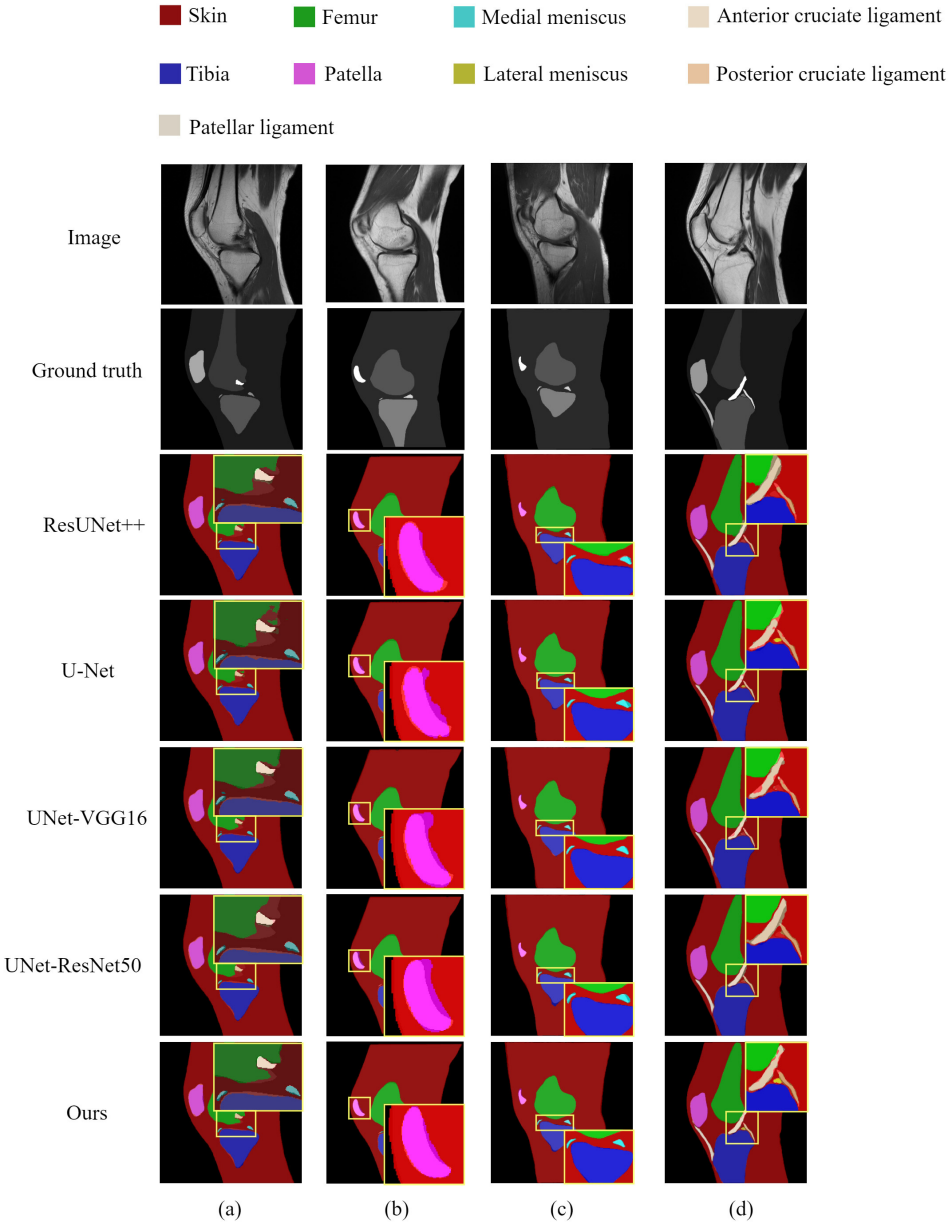
Visual segmentation results of the knee joint generated by the models. Four groups of images **(a–d)** were randomly selected from the test set to compare the segmentation performance of the proposed model with other models.

To provide a visual evaluation and comparison of each model's performance on the knee joint segmentation task, we randomly selected four images from the test set, as shown in Figure 7. On the whole, most models achieved relatively accurate segmentation for larger structures, such as the skin and tibia. However, significant differences were observed in the segmentation of smaller anatomical structures and regions with blurred boundaries. Notably, HASA-ResUNet demonstrated superior results compared to other models. As depicted in Figure 7a, the other four models (ResUNet++, U-Net, UNet-VGG16, and UNet-ResNet50) exhibited evident under-segmentation for the blurred boundary between the femur and the surrounding background, and U-Net also showed discontinuous segmentation. In contrast, our model excelled in defining the boundary with greater precision. Moreover, in Figure 7c, our model achieved almost perfect segmentation of the two medial menisci, outperforming other models. In Figure 7d, ResUNet++, UNet-VGG16, and UNet-ResNet50 failed to segment the lateral meniscus. Although U-Net was able to segment the lateral meniscus, its performance was suboptimal, with inaccurate segmentation of the boundary between the femur and the anterior cruciate ligament. Compared to other models, HASA-ResUNet achieved significantly higher segmentation accuracy, particularly in areas with ambiguous boundaries across various structures. In conclusion, the segmentation results indicate that HASA-ResUNet effectively distinguishes boundaries and mitigates interference, capturing fine details that U-Net and other U-Net variants fail to identify.

**Figure 8:**
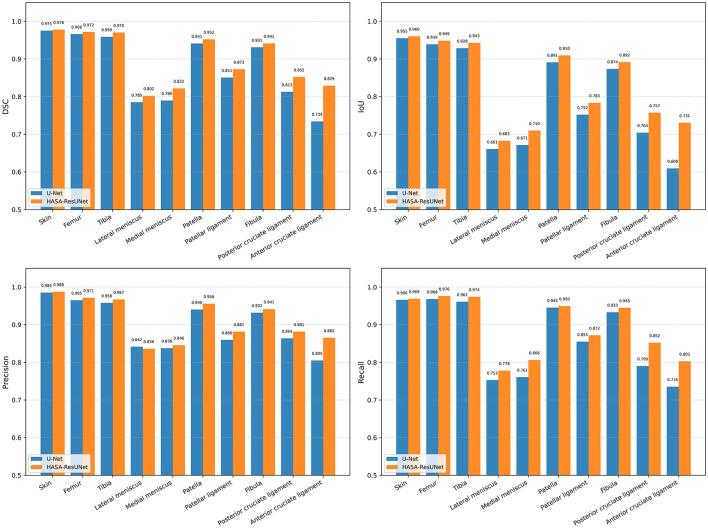
The comparison of HASA-ResUNet with U-Net on different anatomical structures.

Statistics were performed on all categories, as shown in Figure 8. Both models performed best on the bones, with accuracy above 93%. It is worth noting that our model outperforms U-Net in almost all categories, particularly in small structures such as the meniscus and low-frequency categories like the anterior cruciate ligament and the posterior cruciate ligament.

### 3.4 Ablation studies

The ablation experiments aim to explore the efficacy of HFEF and ASA in knee joint segmentation. To this end, we conducted a series of experiments using ResUNet as the baseline model and analyzed the contributions of HFEF and ASA in improving segmentation accuracy. As shown in Table 2. The results demonstrate that the advantages brought by the HFEF and ASA modules are equally important. Compared to ResUNet, the HFEF module improves DSC, IoU, and recall by 1.00%, 1.29%, and 1.42%, respectively. The ASA module further increases DSC, IoU, precision, and recall by 1.07%, 1.41%, 0.13%, and 1.42%, respectively. The integration of the HFEF and ASA modules further enhances DSC, IoU, and recall by 1.55%, 2.07%, and 2.65%. The precision remains roughly at the same level. These results suggest that both HFEF and ASA are effective in improving the performance of knee segmentation.

**Table 2:** Ablation experiment results.

**Methods**	**DSC**	**IoU**	**Precision**	**Recall**
ResUNet	0.8836	0.8110	0.9150	0.8660
ResUNet+HFEF	0.8936	0.8239	0.9147	0.8801
ResUNet+ASA	0.8943	0.8251	**0.9163**	0.8802
ResUNet+HFEF+ASA	**0.8991**	**0.8317**	0.9133	**0.8925**

Bold values indicates the highest metric values.

## 4 Discussion

The main work of this study is to design and integrate the HFEF and ASA modules into a modified ResUNet, and then to visualize and quantitatively evaluate their performance in a multi-class segmentation task of knee MRI images. Currently, several models have been developed for accurately segmenting knee bones and cartilage (27–29). However, to the best of our knowledge, fewer studies have been conducted on total knee segmentation. KOA is a chronic disease that involves multiple structures of the knee joint, therefore, accurate segmentation of the total knee joint is one of the key steps in the intelligent diagnosis and treatment of KOA. The blurred boundaries and severe class imbalance inherent in the complex knee joint structure present significant challenges in segmentation. We used U-Net as the foundation and combined it with ResNet's residual connections to enhance information flow, effectively mitigating the gradient vanishing and information loss problems in deeper U-Net networks. In recent years, integrating attention modules into various networks has become increasingly common. By assigning different weights to different regions, attention mechanisms help the network focus on important areas while suppressing irrelevant or redundant information. In (30), the self-attention mechanism was applied to a computer vision task to capture long-range dependencies, called non-local attention. However, this approach suffers from a problem of low efficiency when the input feature map is very large. Researchers improved the non-local method to enhance efficiency while retaining important information. Fu et al. (31) proposed a Dual Attention Network (DANet), which simulates the semantic interdependence relationships in both spatial and channel dimensions, achieving rich context dependency to perform the scene segmentation task. Inspired by these studies, we introduced two attention modules, HFEF and ASA, to address the limitations of ResUNet in capturing detailed information. By expanding the attention range, our method enhances the model's ability to integrate anatomical structures at different scales, thus enabling it to tackle the complex multi-class segmentation task better.

We compared our method with several state-of-the-art approaches, including the original U-Net model and several modified versions, such as ResUNet++, UNet-VGG16, and UNet-ResNet50. The results, as shown in Table 1, indicate that the best performance is achieved by our proposed HASA-ResUNet method. Despite strong performance on average metrics, we observed that even the best-performing model still exhibited low accuracy in certain structures, such as the menisci and ligaments. This may be due to the small sample size of our dataset, as deep learning models require sufficiently diverse training and validation datasets to capture the features of different structures. Another possible reason is that these structures have unclear boundaries compared to bones, so the manual labeling results may have introduced reader bias. Although the HASA-ResUNet segmentation method has many advantages, there are still some limitations. Our dataset covers the key structures of the knee joint, but it does not include muscles. Furthermore, our method is currently limited to processing normal knee cases and has not been trained with knees with disease. Future work involving training and validation with larger datasets could further improve the model's performance.

## 5 Conclusions

In conclusion, to address the class imbalance and feature extraction challenges in our knee joint dataset, we developed a segmentation network HASA-ResUNet based on hybrid attention mechanism. This model effectively captures details and integrates multi-scale information, improving both small structure accuracy and overall segmentation performance. It enables doctors to segment knee joint structures more accurately and efficiently, providing valuable support for the diagnosis and treatment of KOA. In the future, we aim to conduct further research and collect extensive data to establish a standardized multi-sequence knee joint dataset, benefiting more patients and orthopedic surgeons.

## Data Availability

The datasets presented in this article are not readily available because the data analyzed in this study is subject to the following licenses/ restrictions: data might be requested to authors and it will be sent if authorized by corresponding authorities as they are images of patients. Requests to access the datasets should be directed to tao.meng@imagecore.com.cn.

## References

[1] Atiyah AZ Ali KH. Brain MRI images segmentation based on u-net architecture. IJEEE J. (2021) 18:217. 10.37917/ijeee.18.1.3

[2] Chen H Zhao N Tan T Kang Y Sun C Xie G. Knee bone and cartilage segmentation based on a 3D deep neural network using adversarial loss for prior shape constraint. Front Med. (2022) 9:792900. 10.3389/fmed.2022.792900 35669917 PMC9163741

[3] Chen LC. Rethinking atrous convolution for semantic image segmentation. arXiv [preprint] arXiv:1706.05587. (2017). 10.48550/arXiv.1706.05587

[4] Chen LC Zhu Y Papandreou G Schroff F Adam H. Encoder-decoder with atrous separable convolution for semantic image segmentation. In: Proceedings of the European Conference on Computer Vision. Cham: Springer (2018). p. 801–18.

[5] Felson DT Lawrence RC Dieppe PA Hirsch R Helmick CG Jordan JM. Osteoarthritis: new insights. Part 1: the disease and its risk factors. Ann Intern Med. (2000) 133:635–46. 10.7326/0003-4819-133-8-200010170-00016 11033593

[6] Filippiadis D Charalampopoulos G Mazioti A Alexopoulou E Vrachliotis T Brountzos E. Interventional radiology techniques for pain reduction and mobility improvement in patients with knee osteoarthritis. Diagn Interv Imag. (2019) 100:391–400. 10.1016/j.diii.2019.02.011 30935863

[7] Fu J Liu J Tian H Li Y Bao Y Fang Z Lu H. Dual attention network for scene segmentation. In: Proceedings of the IEEE/CVF Conference on Computer Vision and Pattern Recognition. Long Beach, CA: IEEE (2019). p. 3146–3154. 10.1109/CVPR.2019.00326

[8] Gaj S Yang M Nakamura K Li X. Automated cartilage and meniscus segmentation of knee MRI with conditional generative adversarial networks. Magn Reson Med. (2020) 84:437–49. 10.1002/mrm.28111 31793071

[9] Gan H-S Ramlee MH Wahab AA Lee Y-S Shimizu A. From classical to deep learning: review on cartilage and bone segmentation techniques in knee osteoarthritis research. Artif Intellig Rev. (2021) 54:2445–94. 10.1007/s10462-020-09924-4

[10] He K Zhang X Ren S Sun J. Deep residual learning for image recognition. In: Proceedings of the IEEE Conference on Computer Vision and Pattern Recognition. Las Vegas, NV: IEEE (2016). p. 770–778.

[11] Heimann T Morrison BJ Styner MA Niethammer M Warfield S. Segmentation of knee images: a grand challenge. In: Proc. MICCAI Workshop on Medical Image Analysis for the Clinic. Beijing: MICCAI (2010).

[12] Huang M Schweitzer ME. The role of radiology in the evolution of the understanding of articular disease. Radiology. (2014) 273:S1–S22. 10.1148/radiol.14140270 25340431

[13] Jha D Smedsrud PH Riegler MA Johansen D De Lange T Halvorsen P Johansen HD. Resunet++: An advanced architecture for medical image segmentation. In: 2019 IEEE International Symposium on Multimedia (ISM). San Diego, CA: IEEE (2019). p. 225–2255.

[14] Khan S Azam B Yao Y Chen W. Deep collaborative network with alpha matte for precise knee tissue segmentation from MRI. Comput Methods Programs Biomed. (2022) 222:106963. 10.1016/j.cmpb.2022.106963 35752117

[15] Kumar D Gandhamal A Talbar S Hani AFM. Knee articular cartilage segmentation from MR images: a review. ACM Comp Surv (CSUR). (2018) 51:1–29. 10.1145/3230631

[16] Li Z Kamnitsas K Glocker B. Analyzing overfitting under class imbalance in neural networks for image segmentation. IEEE Trans Med Imaging. (2020) 40:1065–77. 10.1109/TMI.2020.3046692 33351758

[17] Liu F Zhou Z Jang H Samsonov A Zhao G Kijowski R. Deep convolutional neural network and 3D deformable approach for tissue segmentation in musculoskeletal magnetic resonance imaging. Magn Reson Med. (2018) 79:2379–91. 10.1002/mrm.26841 28733975 PMC6271435

[18] Meng X Zhu L Han Y Zhang H. We need to communicate: communicating attention network for semantic segmentation of high-resolution remote sensing images. Remote Sens. 15:3619. 10.3390/rs15143619

[19] Milletari F Navab N Ahmadi SA. V-net: Fully convolutional neural networks for volumetric medical image segmentation. In: 2016 Fourth International Conference on 3D Vision (3DV). Stanford, CA: IEEE (2016). p. 565–571.

[20] Morales Martinez A Caliva F Flament I Liu F Lee J Cao P. Learning osteoarthritis imaging biomarkers from bone surface spherical encoding. Magn Reson Med. (2020) 84:2190–203. 10.1002/mrm.28251 32243657 PMC7329596

[21] Nasser Y Jennane R Chetouani A Lespessailles E El Hassouni M. Discriminative regularized auto-encoder for early detection of knee osteoarthritis: data from the osteoarthritis initiative. IEEE Trans Med Imaging. (2020) 39:2976–84. 10.1109/TMI.2020.2985861 32286962

[22] Prasoon A Petersen K Igel C Lauze F Dam E Nielsen M. Deep feature learning for knee cartilage segmentation using a triplanar convolutional neural network. In: International Conference on Medical Image Computing and Computer-Assisted Intervention. Cham: Springer (2013). p. 246–253. 10.1007/978-3-642-40763-5_31 24579147

[23] Pravitasari AA Iriawan N Almuhayar M Azmi T Irhamah I Fithriasari K. UNet-VGG16 with transfer learning for MRI-based brain tumor segmentation. Telecommun Comp Electron Cont. (2020) 18:1310–8. 10.12928/telkomnika.v18i3.14753

[24] Ronneberger O Fischer P Brox T. U-Net: Convolutional networks for biomedical image segmentation. In: Medical Image Computing and Computer-Assisted Intervention-MICCAI 2015: 18th International Conference. Munich: Springer. (2015). p. 234–241.

[25] Sun K Xiao B Liu D Wang J. Deep high-resolution representation learning for human pose estimation. In: Proceedings of the IEEE/CVF Conference on Computer Vision and Pattern Recognition. Long Beach, CA: IEEE (2019). p. 5693–5703.

[26] Timmins KA Leech RD Batt ME Edwards KL. Running and knee osteoarthritis: a systematic review and meta-analysis. Am J Sports Med. (2017) 45:1447–57. 10.1177/0363546516657531 27519678

[27] Wang X Girshick R Gupta A He K. Non-local neural networks. In: Proceedings of the IEEE Conference on Computer Vision and Pattern Recognition. Salt Lake City, UT: IEEE (2018). p. 7794–7803.

[28] Woo B Engstrom C Baresic W Fripp J Crozier S Chandra SS. Automated anomaly-aware 3D segmentation of bones and cartilages in knee MR images from the osteoarthritis initiative. Med Image Anal. (2024) 93:103089. 10.1016/j.media.2024.103089 38246088

[29] Zhang H Zu K Lu J Zou Y Meng D. EPSANet: an efficient pyramid squeeze attention block on convolutional neural network. In: Proceedings of the Asian Conference on Computer Vision. Cham: Springer (2022). p. 1161–1177.

[30] Zhou Z Zhao G Kijowski R Liu F. Deep convolutional neural network for segmentation of knee joint anatomy. Magn Reson Med. (2018) 80:2759–70. 10.1002/mrm.27229 29774599 PMC6342268

[31] Zhuang Z Si L Wang S Xuan K Ouyang X Zhan Y. Knee cartilage defect assessment by graph representation and surface convolution. IEEE Trans Med Imag. (2022) 42:368–79. 10.1109/TMI.2022.3206042 36094985

